# Decomposing international gender test score differences

**DOI:** 10.1186/s12651-018-0246-8

**Published:** 2018-11-19

**Authors:** Farzana Munir, Rudolf Winter-Ebmer

**Affiliations:** 1 Bahauddin Zakariya University, Multan, Pakistan; 2 Christian Doppler Laboratory Aging, Health and the Labor Market, IZA, CEPR and IHS, Johannes Kepler University, Linz, Austria

**Keywords:** Gender gap, Test scores, PISA, Mathematics, Reading, I23, I24, J16

## Abstract

In this paper, we decompose worldwide PISA mathematics and reading scores. While mathematics scores are still tilted towards boys, girls have a larger advantage in reading over boys. Girls’ disadvantage in mathematics is increasing over the distribution of talents. Our decomposition shows that part of this increase can be explained by an increasing trend in productive endowments and learning productivity, although the largest part remains unexplained. Countries’ general level of gender (in)equality also contributes to girls’ disadvantage. For reading, at the upper end of the talent distribution, girls’ advantage can be fully explained by differences in learning productivity, but this is not so at lower levels.

## Introduction

Consensus exists regarding significant gender test score differences in schools. Boys typically excel in mathematics and science whereas girls score better in reading and literacy subjects (e.g., Turner and Bowen 1999; Halpern et al. 2007; Ceci et al. 2009). Although girls have somewhat caught up in mathematics (Hyde and Mertz 2009), differences remain. On the other hand, there is evidence of more men or boys at the upper end of the education or professional distribution (Machin and Pekkarinen 2008), which could be attributed to the larger variance of test scores for boys. The magnitude, spread and practical significance of gender differences in educational outcomes have remained a topic of concern. This concern is important, because gender disparities in achievement at an earlier stage, particularly at the upper ends of the distribution, may impact career selection and educational outcomes at a later stage.

The previous literature mostly examined mean differences (Fryer and Levitt 2010), while quantile regressions do exist for some countries (Gevrek and Seiberlich 2014; Sohn 2012; Thu Le and Nguyen 2018): providing evidence for Turkey, Korea and Australia, respectively. Two possible arguments have been suggested for these gender gaps, one biological or natural (Benbow and Stanley 1980; Geary 1998) and the other environmental, including family, institutional, social, and cultural influences (e.g., Fennema and Sherman 1978; Parsons et al. 1982; Levine and Ornstein 1983; Guiso et al. 2008; Pope and Sydnor 2010; Nollenberger et al. 2016). Recent studies looked at the impact of culture: Nollenberger et al. (2016) look at immigrants in the U.S. to explain whether gender-related culture in the home country can explain differences in mathematics scores; similarly Guiso et al. (2008) look at gender differences in 35 countries PISA mathematics scores.

The present study looks at mathematics and reading scores for all countries included in the OECD’s PISA test and tries to decompose these score differences at different percentiles of the distribution through natural and environmental factors that influence the students’ mathematics and reading test scores. This decomposition research is guided by the Juhn et al. (1993) decomposition model, which extends the usual Blinder–Oaxaca decomposition by taking into account the residual distribution. Following this method, this study will decompose test score gaps between males and females to analyze how much of the test score gap can be “predicted” by observable differences across students in determining the test score production function and inequality within these classifications.

In this study, we employed international PISA data to examine test score differences between boys and girls worldwide, focusing on the differences at different quantiles of the distribution. PISA has the advantage of covering various personal, family, school system, and societal background characteristics, which enables decomposing potential differences into effects due to different endowments, institutional settings, and the productivity of learning in different situations. We adopted a decomposition following Juhn et al. (1993), which enabled us to decompose test score differentials into endowment, productivity, and unobservable components.

Our decomposition for score differentials in mathematics shows that part of the increasing disadvantage of girls over the distribution of talent can be explained by an increasing trend in productive endowments and learning productivity, although the largest part remains unexplained. Countries’ general level of gender (in)equality also contributes to girls’ disadvantage. For reading, at the upper end of the talent distribution, girls’ advantage can be fully explained by differences in learning productivity, but this is not so at lower levels. Our contribution to the literature lies in an extension of quantile regression results to practically all PISA countries, to an inclusion of country-specific gender-related variables and to an application of the Juhn, Murphy and Pierce analysis, which extends a simple decomposition to take the residual distribution into account.

The remainder of the paper is organized as follows: The next section describes the PISA database, its features and other data sources used in the study. Section 3 discusses the estimation strategy used in this paper and structures the econometric model based upon the Juhn, Murphy and Pierce decomposition method. Section 4 presents results on test score inequality for our dispersion analysis. Section 5 concludes.

## Data

This paper uses the micro data of the Program of International Student Assessment (PISA) 2012 as well as data on per capita GDP (PPP), gender equality, and government expenditure on education to analyze the decomposition of gender differences in test scores. Combining the available data, the dataset contains information on 480,174 students in 65 countries pertaining to mathematics and reading literacy.

### PISA data

PISA is a cross-national study created by the Organization for Economic Co-operation and Development (OECD) to assess students’ ability in mathematics, reading, science, and problem solving. Since its launch in 2000, the assessment is conducted on a triennial basis. The main advantage of the program is its international comparability, as it assesses students’ ability based on a cohort of students of the same age. Moreover, there is a large volume of background information of students and schools, which may help to put student assessment into perspective. The assessment in each wave focuses on one particular subject,^1^ and tests other main areas as well. In our analysis, we employed data from the 2012 PISA wave that focused on performance in mathematics.

The PISA 2012 dataset covers the test score performance of students from 34 OECD and 31 non-OECD countries, which includes approximately 510,000 students aged 15 or 16 years. The dataset includes a number of demographic and socioeconomic variables for these students. The instrument was paper-based and comprised a mixture of text responses and multiple-choice questions. The test is completed in 2 h. The questions are organized in groups based on real life situations. A stratified sampling design was used for this complex survey, and at least 150 schools were selected^2^ in each country and 35 students randomly selected in each school to form clusters. Because of potential sample selection problems, weights were assigned to each student and school. The PISA test scores are standardized with an average score of 500 points and standard deviation of 100 points in OECD countries. In the PISA 2012 test, the final proficiency estimates were provided for each student and recorded as a set of five plausible values.^3^ In this study, we used the first plausible value as a measure of student proficiency.^4^

In 2012, Shanghai scored best and remained at the top with 613 PISA points in mathematics, followed by Hong Kong, Japan, Taiwan, and South Korea, all high-performing East Asian countries. Among the European countries, Liechtenstein and Switzerland demonstrated the best performance, followed by the Netherlands, Estonia, Finland, Poland, Belgium, Germany, and Austria with slightly lower figures. On average, the mean score in mathematics was 494 and 496 for reading in OECD countries. The UK, Ireland, New Zealand, and Australia were close to the OECD average, while the USA scored lower than the OECD average with 481 PISA points.

Since the primary concern of this study is to explore the differences in mathematics and reading test scores between male and female students, the dependent variable is the student test score in PISA 2012. The rich set of covariates includes five characteristics, namely individual characteristics of the students, their family characteristics, school characteristics, student’s beliefs or perceptions about learning, and country characteristics. Table 2 provides a description of all variables from the PISA data used in this study.

In the survey data, the probability that individuals will be sampled is assumed dependent on the survey design. To take into account this feature, students’ educational production functions were estimated using survey regression methods. This allowed us to include student weights and school clusters depending on the sampling probabilities and within standard errors respectively in our analysis.

**Fig. 1:**
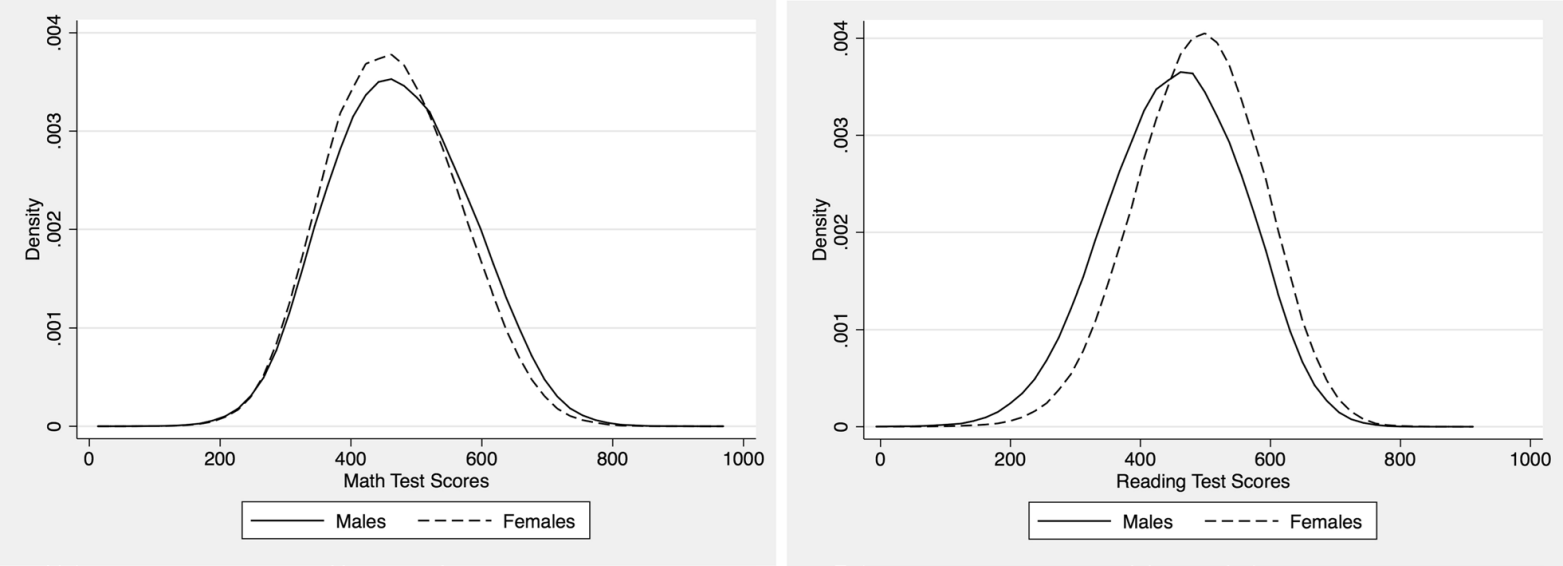
Kernel density estimation of PISA test score 2012 in mathematics and reading

Non-parametric kernel density estimates for the distribution of the entire sample of students’ test score achievements by gender are presented in Fig. 1. The left and right panels of Fig. 1 display kernel density estimates for mathematics and reading test performances respectively. Males’ test scores in mathematics are on average higher than those for females, whereas females on average score better than males for reading. Regarding the spread of the curves, it is narrow and highly concentrated around the mean for females compared to the relatively wider distribution of males both in mathematics and reading test scores.

### Level of development, education expenditure, and gender equality data

To consider the country’s level of development in this analysis, we employed the data on GDP per capita (measured in purchasing power parity (PPP)) from the World Development Indicators 2012. Data on education expenditure was derived from the Human Development Report 2013, United Nations Development Program, while data for Jordan, Shanghai, and Macao were obtained from the World Bank database.

To explore the cultural role related to gender equality, following Guiso et al. (2008), we employed the Gender Gap Index (GGI) by the World Economic Forum (Hausmann et al. 2013). The Global Gender Gap Index was first introduced in 2006, which by that time was published annually by the World Economic Forum. GGI shows the ranking of countries based on the average of four sub indices,^5^ namely economic, political, health, and educational opportunities provided to females. A GGI of 1 reflects full gender equality and 0 total gender inequality. The top five countries in the 2012 GGI ranking were Iceland (0.86), Finland (0.85), Norway (0.84), Sweden (0.82), and Ireland (0.78). It is important to note that GGI data is only available for whole countries^6^ and not for participating economic regions in the PISA 2012 dataset (e.g., Hong Kong, Macao, and Shanghai), Furthermore, it does not seem reasonable that data for whole countries can be representative of the relevant economic regions. These regions were eliminated from the data set.^7^

## Estimation strategy

In general, decomposition approaches follow the standard partial equilibrium approach in which observed outcomes of one group (i.e., gender, region, or time period) can be used to construct various counterfactual scenarios for the other group. Besides this, decompositions also provide useful indications of particular hypotheses to be explored in more detail (Fortin et al. 2011).

Originally, decomposition methods were proposed by Oaxaca (1973) and Blinder (1973) for decomposing differences in the means of an outcome variable. The Juhn et al. (JMP) (1993) decomposition method extends the Oaxaca/Blinder decomposition by considering the residual distribution.^8^ We show this decomposition following the description of Sierminska et al. (2010) as follows:

1 $${\text{Y}}_{\text{j}} = {\text{X}}_{\text{j}}\upbeta_{\text{j}} +\upvarepsilon_{\text{j}}$$

where Y_j_ are the test scores for j=M, W (men and women respectively), X_j_ are observables, β_j_ are the vectors of the estimated coefficients, and ε_j_ are the residuals (unobservables, i.e., unmeasured prices and quantities).

If F_j_(.) denotes the cumulative distribution function of the residuals for group j, then the residual gap consists of two components: an individual’s percentile in the residual distribution p_i_, and the distribution function of the test score equation residuals F_j_(.). If p_ij _= F_j_(ε_ij_|x_ij_) is the percentile of an individual residual in the residual distribution of model I, by definition we can write the following:

2 $$\upvarepsilon_{\text{ij}} = {\text{F}}_{i}^{ - 1} \left( {{\text{p}}_{\text{ij}} |{\text{x}}_{\text{ij}} } \right)$$

where F_j_^−1^(.) is the inverse of the cumulative distribution (e.g., the average residual distribution over both samples) and $\overline{\beta }$ an estimate of benchmark coefficients (e.g., the coefficients from a pooled model over the whole sample).

Using this framework, we can construct hypothetical outcome distributions with any of the components held fixed. Thus, we can determine:

1. Hypothetical outcomes with varying quantities between the groups and fixed prices (coefficients) and a fixed residual distribution as
  3 $${\text{y}}_{\text{ij}}^{(1)} = {\text{x}}_{\text{ij}} \overline{\beta } + {\text{ F}}_{\text{i}}^{ - 1} \left( {{\text{p}}_{\text{ij}} |{\text{ x}}_{\text{ij}} } \right)$$

2. Hypothetical outcomes with varying quantities and varying prices and fixed residual distribution as
  4 $${\text{y}}_{\text{ij}}^{(2)} = {\text{x}}_{\text{ij}}\upbeta_{\text{j}} + {\text{ F}}_{\text{i}}^{ - 1} \left( {{\text{p}}_{\text{ij}} |{\text{x}}_{\text{ij}} } \right)$$

3. Outcomes with varying quantities, varying prices, and a varying residual distribution^9^ as
  5 $${\text{y}}_{\text{ij}}^{(2)} = {\text{ x}}_{\text{ij}}\upbeta_{\text{j}} + {\text{F}}_{\text{i}}^{ - 1} \left( {{\text{p}}_{\text{ij}} |{\text{ x}}_{\text{ij}} } \right)$$

Let a capital letter stand for a summary statistic of the distribution of the variable denoted by the corresponding lower-case letter. For instance, Y may be the mean or interquartile range of the distribution of y. The differential Y_M_–Y_W_ can then be decomposed as follows:

6 $$\begin{aligned} {\text{Y}}_{\text{M}} - {\text{Y}}_{\text{W}} & = \left[ {{\text{Y}}_{\text{M}}^{( 1)} - {\text{Y}}_{\text{W}}^{( 1)} } \right] + \left[ {\left( {{\text{Y}}_{\text{M}}^{( 2)} - {\text{Y}}_{\text{W}}^{( 2)} } \right){-}\left( {{\text{Y}}_{\text{M}}^{( 1)} - {\text{Y}}_{\text{W}}^{( 1)} } \right)} \right] \, \\ & \, \, \, + \left[ {\left( {{\text{Y}}_{\text{M}}^{( 3)} - {\text{Y}}_{\text{W}}^{( 3)} } \right){-}\left( {{\text{Y}}_{\text{M}}^{( 2)} - {\text{Y}}_{\text{W}}^{( 2)} } \right)} \right] \, \\ & = {\text{T }} = {\text{Q}} + {\text{P}} + {\text{U}} \\ \end{aligned}$$

where T is the total difference, Q can be attributed to differences in observable endowments, P to differences in the productivity of observable contributions to test scores, and U to differences in unobservable quantities and prices. This last component not only captures the effects of unmeasured prices and differences in the distribution of unmeasured characteristics (e.g., one of the unmeasured characteristics is more important for men and women for generating test scores), but also measurement error.

The major advantage of the JMP framework is that it enables us to examine how differences in the distribution affect other inequality measures and how the effects on inequality differ below and above the mean.

## Estimation results

### Descriptive statistics

Table 4 contains the descriptive statistics on all the variables used in this microanalysis of the PISA, 2012 dataset. The descriptive statistics are displayed by gender and by OECD and non-OECD countries separately. We imputed missing data for the variable ‘age’ and for some other variables^10^ in the schooling vector by using the mean imputation method.

Table 4 shows that in OECD countries, students on average, scored 42.12 and 46.1 points more in mathematics and reading, respectively than non-OECD countries. On average, OECD girls have fallen behind OECD boys by 5.4 points in mathematics scores and 9 points in reading scores, while, non-OECD girls remain 3.5 PISA points behind non-OECD boys in mathematics and 6.5 in reading.

In order to examine whether or not a gender difference within PISA is statistically significant at the 1%, 5% and 10% levels, we also calculated the mean difference between the girls’ and boys’ scores.^11^ It shows that significant mean differences across gender (based on the OECD and non-OECD grouping) exist for almost all variables.

### PISA score in mathematics

**Fig. 2:**
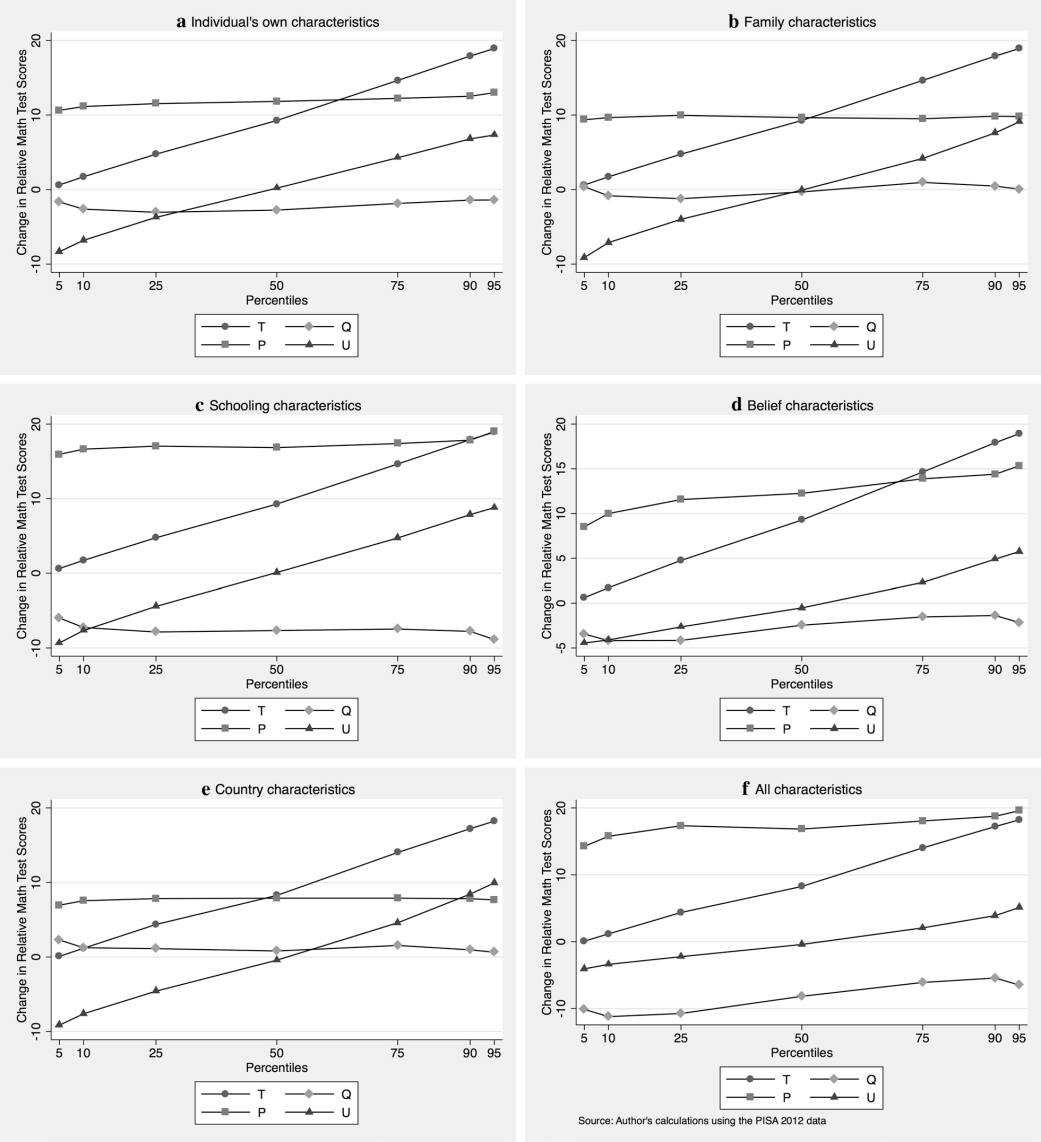
Juhn–Murphy–Pierce decomposition of relative mathematics test scores by percentile, 2012, *T* total differential, *Q* endowments, *P* productivity, *U* unobservables, **a**–**e** provide decompositions using only a subset of variables; **f** uses all available variables

Decomposition results for the mathematical test scores following JMP are depicted in Fig. 2. Positive results indicate females’ disadvantage. In Fig. 2, we include a varying set of control variables: individual’s characteristics, family characteristics, school characteristics, characteristics of beliefs about the learning process, and country characteristics. Panels A–E provide the decomposition results including only one of these lists of covariates. Panel F shows a decomposition using all available covariates together. Male–female test score differences are shown at various percentiles: 5th, 10th, 25th, 50th, 75th, 90th, and 95th. Table 6 in Appendix provides the numerical results.

In general, a strong upward trend in the total male–female test score differential (T) is evident. While there is (almost) no difference for the lowest percentiles, the female disadvantage in mathematical competence increases almost linearly to around 20 PISA points at the 95th percentile. As good mathematical knowledge, particularly at the upper percentiles, is especially valuable for getting a good job (Athey et al. 2007), it is important to explore this issue. This total (T) effect will be decomposed into an effect due to differences in observables (Q), in a productivity-effect (P) on the learning productivity of these observables, and finally, an unobservable (U) rest.

Looking first at Panel F—including all characteristics, this upward trend in mathematical test score differences (T) cannot easily be explained by one factor. Unobservables demonstrate a clear upward trend, but observables and productivity effects do so at a somewhat lower level. We now examine individual contributions of individual versus school characteristics. Here, decomposing the contribution of unobservables (U) in Panels A–E does not make sense, because even if the individual contributions are orthogonal, the unobservable trends measure mainly the impact of omitted variables.

Turning to the contribution of observables (Q) towards mathematical competence, the endowment effect, Panel F indicates a negative endowment effect. In other words, females typically enjoy better endowments: around 10 PISA points at lower percentiles down to 5 PISA points at higher levels. These advantages stem from better female endowments in terms of schooling characteristics and beliefs. The slight upward trend in the contribution of observables in Panel F can mainly be attributed to an upward trend in observables in belief characteristics.

What is the contribution of learning productivity (P)? Panel F shows that the learning productivity of females increases the male–female test score gap for all percentiles, but the effect is slightly higher for higher percentiles. Panels A–E indicate similar productivity disadvantages for all included lists of characteristics.

To examine the contribution of individual variables in more detail, we performed the following quantitative exercise: increase, in turn, one of the variables in the model by one standard deviation and calculate the impact on the PISA score for males and females (Table 1). Starting with variables that will increase the male test score advantage, the number of female students in a classroom has the largest positive effect. Increasing the female share by one standard deviation increases the male–female test score differential by 8.8 PISA points. This is contrary to the results of Gneezy et al. (2003), who found that more female peers in schools increases the mathematical competence of females. Other strong pro-male variables are students’ beliefs such as perseverance, success, or a career or job motive. Factors that reduce the male–female gap are subjective norms, public schools, more studying outside school, better education of the mother, and mothers who work more. Interestingly, countries where the GGI is more favorable towards women have lower male–female PISA score differences. This is in contrast to simple correlations by Stoet and Geary (2013), which did not reveal any correlation between PISA gender differentials and the GGI.

**Table 1:** Ceteris–paribus shifts in math and reading test scores due to a one standard deviation shift in individual variables

	Mathematics			Reading		
	Male	Female	Gender score difference	Male	Female	Gender score difference
*Individual characteristics*						
Age	1.001	0.930	0.071	0.731	0.775	− 0.567
Grade	11.66	9.950	1.71	12.67	10.24	2.43
Country of birth	1.675	1.577	0.098	1.235	1.098	0.137
*Family characteristics*						
Mother’s education	4.30	6.09	− 1.79	4.706	5.947	− 1.241
Father’s education	5.414	5.457	− 0.043	4.180	3.976	0.204
Mother’s work	4.217	5.763	− 1.546	3.605	5.354	− 1.749
Father’s work	5.841	5.467	0.374	5.540	4.896	0.644
Family structure	1.734	1.178	0.556	0.930	− 0.106	1.036
Language	2.401	0.856	1.545	6.44	5.276	1.164
Home possession	16.89	17.83	− 0.94	14.98	17.51	− 2.53
*Schooling characteristics*						
Public schools	− 3.897	− 1.769	− 2.128	− 7.069	− 2.88	− 4.189
	6.370	7.563	− 1.193	5.502	6.234	− 0.732
Class size	9.425	9.122	0.303	10.44	7.932	2.508
Quality of physical infrastructure	2.904	2.65	0.254	2.183	1.534	0.649
Percentage of girls at school	7.983	− 0.872	8.855	8.807	1.667	7.14
Certified teachers	7.697	9.528	− 1.831	6.796	7.164	− 0.368
Teacher–student ratio	− 3.570	− 4.763	1.193	− 1.818	− 2.858	1.04
Teacher–student relations	− 1.409	− 0.218	− 1.191	− 1.580	− 1.120	− 0.46
*Belief characteristics*						
Difference in test efforts	− 3.565	− 2.083	− 1.482	− 5.635	− 3.837	− 1.798
Out of school study hours	1.586	5.825	− 4.239	0.236	3.810	− 3.574
Perseverance	9.765	6.977	2.788	8.79	6.136	2.654
Success	16.52	10.85	5.67	11.85	6.055	5.795
Career motive	12.52	10.06	2.46	7.424	5.476	1.948
Job motive	− 2.88	− 4.589	1.709	− 8.765	− 9.541	0.776
Subjective norms	− 12.40	− 9.155	− 3.245			
*Country characteristics*						
GDP	− 0.342	0.963	− 1.305	0.976	0.723	0.253
GGI	− 0.908	1.507	− 2.415	0.826	0.621	0.205
Gender ratio at PISA	− 12.21	− 10.37	− 1.84	− 7.641	− 7.302	− 0.339
Education expenditure	11.47	11.02	0.45	12.06	12.74	− 0.68

Gender score inequality is calculated by subtracting the female scores from male scores where positive values are indicating the gender inequality towards females. Male and female test scores are calculated on the basis that one standard deviation increase in particular characteristic e.g. age is associated with an increase of 0.071 score points in math gender score gap

### PISA scores for reading

**Fig. 3:**
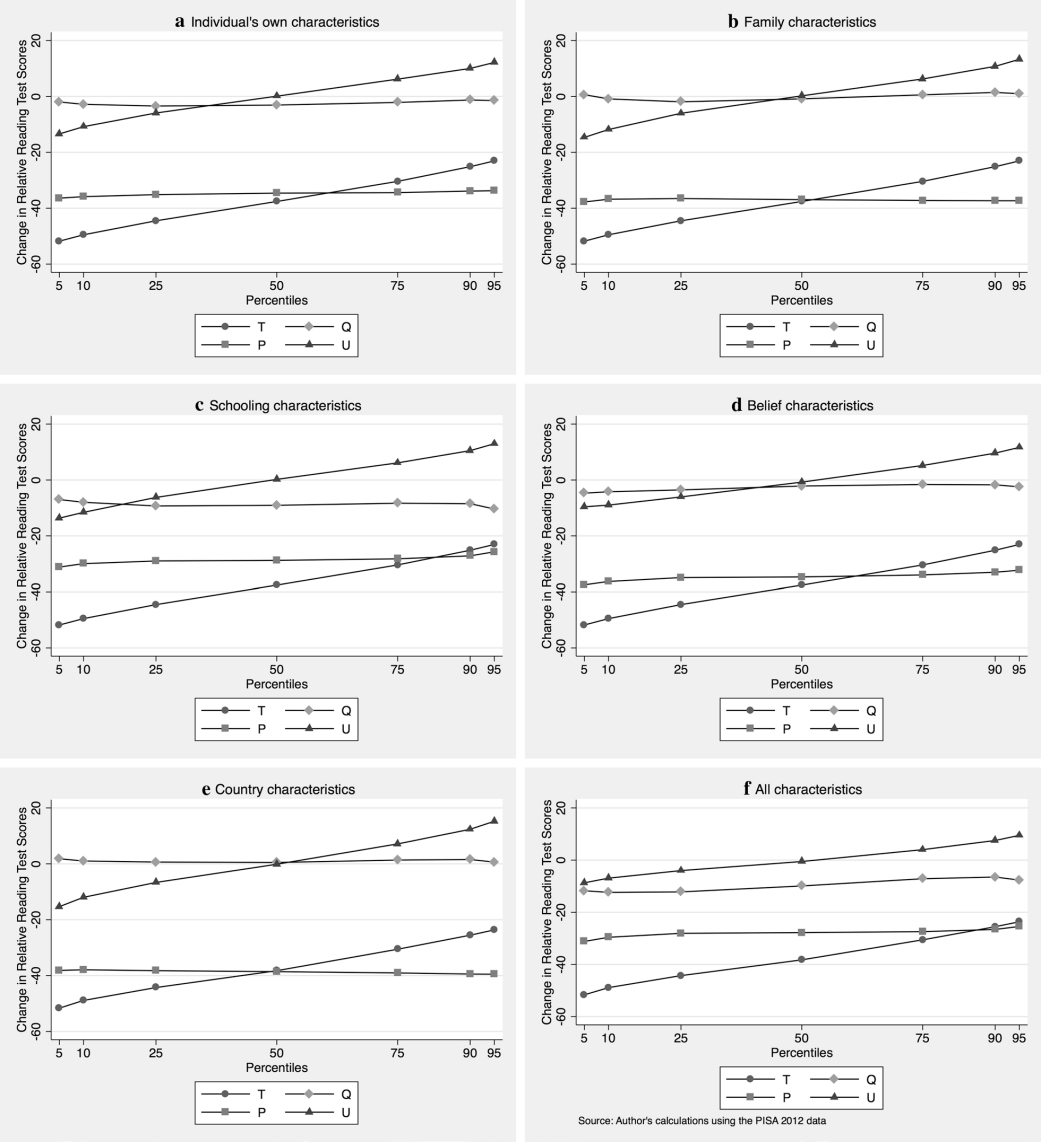
Juhn–Murphy–Pierce decomposition of relative reading test scores by percentile, 2012, *T* total differential, *Q* endowments, *P* productivity, *U* unobservables, **a**–**e** provide decompositions using only a subset of variables; **f** uses all available variables

An equivalent analysis was conducted for reading, as shown in Fig. 3. Panel F shows the JMP decomposition when all control variables are included. In contrast to mathematics, a continuous advantage of girls over boys is evident. In particular, there is a large disadvantage for boys at the lower end of the distribution: at the 5th and 10th percentile, boys score almost one half standard deviation (50 PISA points) less than girls. Torppa et al. (2018) investigate this for an extension of Finnish PISA data and find that general reading fluency (speed) is the main explanation for this difference, whereas other indicators like mastery orientation, homework activity or leisure book reading frequency are not very influential.

On the other hand, similar to mathematics, the total advantage of girls (T) diminishes from around 50 PISA points at the lowest percentiles to about 20 PISA points at the highest.^12^ Decomposing that, at the highest percentile levels, this male–female differential is fully explained by productivity differentials (P), less so at lower percentiles. There is a contribution of observables (Q): the endowment of students contributes between 6 and 12 PISA points towards this female advantage. Finally, the contribution of unobservables (U) is mixed, increasing between − 9 to + 9 PISA points.

Which factors are responsible for this difference? Our detailed analysis of the causes in Panels A–E in Fig. 3 indicates that endowment differences (Q) are strongest for schooling characteristics. Schooling characteristics, considered separately, explain between 7 and 10 PISA points, while the contributions of other domains are minor.

On the other hand, there is a large productivity (P) contribution in all separately considered domains. They are particularly high in the family, individual, belief, and country domains.

Regarding the contributions of individual items (Table 1), those favorable for boys are the percentage of girls in a classroom, success motivation, and class size. Factors favorable for girls are public schools and the amount of studying time out of school. Interestingly, a country’s GGI has no effect on the reading differential between boys and girls.

## Conclusion

In this paper, we provided a decomposition of PISA mathematics and reading scores worldwide. Our contribution to the literature lies in an extension of quantile regression results to practically all PISA countries, to an inclusion of country-specific gender-related variables and to an application of Juhn et al. (1993) analysis, which extends a simple decomposition to take the residual distribution into account.

While mathematics scores are still tilted towards boys, girls have a larger advantage in reading over boys. This advantage is particularly large for low-achieving individuals. Our analysis shows that over the distribution of talent, boys’ scores increase more than girls—for both mathematics and reading: thus—at the highest percentiles—we see a smaller reading advantage for girls as well as a large advantage of boys in mathematics.

Our decomposition shows that part of this increase can be explained by an increasing trend in productive endowments and learning productivity, but the largest part remains unexplained. Countries’ general level of gender (in)equality also contributes towards girls’ disadvantage. For reading, at the upper end of the talent distribution, girls’ advantage can be fully explained by differences in learning productivity, although this is not so at lower levels. Education policy trying to reduce these gender differences must target high-performing females in their efforts in mathematics and science, and must be concerned by low-achieving boys who lag in reading and verbal expressiveness.

## Appendix

See Tables 2, 3, 4, 5, 6, and 7.

**Table 2:** Variables’ description (PISA 2012). Sources: (1) PISA Technical Report 2012; (2) PISA Data Analysis Manual SPSS 2009 (Second Edition)

Variable	Definition
*Students’ own characteristics*	
Age	Age of student was calculated as the difference between the year and month of the testing and the year and month of the students’ birth
Grade	The relative grade index was computed to capture between the country variation. It indicates whether students are below or above the model grade in a country (model grade having value “zero”)
Country of birth	According to the PISA, students’ are distinguished by country of birth to take into account their immigrant status 1. “Native students”, students born in the country of assessment with at least one parent born in the country of assessment 2. “Second-generation students”, students born in the country with both parents foreign-born 3. “First-generation students, where foreign-born students have foreign-born parents In this study, the variable for country of birth only differentiate that the students are “native” or “others”
*Family characteristics*	
Educational level of mother and father	Educational levels were classified using ISCED (OECD 1999) that is International Standard Classification of Education. Indices were constructed for the following categories 1. “0” for “None 2. “1” for “primary education” 3. “2” for “lower secondary” 4. “3” for “upper secondary” 5. “4” for “post secondary” 6. “5” for “vocational tertiary” 7. “6” for “theoretical tertiary (or post graduate)”
Occupational status of parents	Parents’ job status is closely linked to socio-economic status that can cause large gaps in performance between students. Students reported their mothers’ and fathers’ current job status either as “full or part time working” or they hold another job status (i.e. home duties, retired etc.)
Family structure	An index was formed on the basis of the family structure with the following categories 1. “1” if “single parent family” (students living with one of the following: mother, father, male guardian, female guardian) 2. “2” if “two parent family” (students living with a father or step/foster father and a mother or step/foster mother) 3. “3” if students do not live with their parents
Language spoken at home	An international comparable variable is derived from the information (containing a country-specific code for each language) with the following categories 1. Language at home is the same as the language of assessment for the student 2. Language at home is another language
Home possession	Home possession is the summary index of 23 household items, mainly related to possession of books and things necessary to have a profound study
*Schooling characteristics*	
School category	Schools are classified as either public or private according to whether a private entity or a public agency has the ultimate power to make decisions concerning its affairs
School autonomy	Twelve items measuring school autonomy were asked that includes (a) Selecting teachers for hire, (b) Firing teachers, (c) Establishing teachers’ starting salaries, (d) Determining teachers’ salary increases, (e) Formulating the school budget, (f) Deciding on budget allocations within the school, (g) Establishing student disciplinary policies, (h) Establishing student assessment policies, (i). Approving students for admission to the school, (j) Choosing which textbooks are used, (k) Determining course content, and (k). Deciding which courses are offered. Five response categories were used and principals were asked to tick as many categories as appropriate, that are 1. Principal 2. Teachers 3. School governing board 4. Regional education authority 5. National education authority
Class size	The average class size was derived from one of the nine possibilities ranging from “15 students or fewer” to “more than 50 students” for the average class size of the test language in the sampled schools. The mid point of each response category was used for class size, resulting a value of 13 for the lowest category, and a value of 53 for the highest
Quality of physical infrastructure	The index concerning the quality of physical infrastructure was computed on the basis of three items measuring the principals’ perceptions of potential factors hindering instruction at school that are (a) Shortage or inadequacy of school buildings and grounds, (b) Shortage or inadequacy of heating/cooling and lighting systems, and (c) Shortage or inadequacy of instructional space (i.e. classrooms). All items were reversed for scaling
Proportion of girls enrolled at school	Proportion is based on the enrollment data provided by the principal, calculated by dividing the number of girls by the number of girls and boys at a school
Proportion of fully certified teachers	The proportion was calculated by dividing the number of fully certified teachers by the total number of teachers
Student–teacher ratio	The student–teacher ratio is obtained by dividing the school size by the total number of teachers. The number of part–time teachers was weighted by 0.5 and the number of full-time teachers was weighted by 1.0 in the computation of this index
Teacher–student relations	The index of teacher–student relations is derived from students’ view that to what extent do you agree with the following statements”: (i) Students get along well with most of my teachers; (ii) Most teachers are interested in students’ well-being; (iii) Most of my teachers really listen to what I have to say; (iv) if I need extra help, I will receive it from my teachers; and (v) Most of my teachers treat me fairly. Higher values on this index indicate positive teacher–student relations
*Students’ perceptions or beliefs about learning*	
Difference in test effort	To compare the students’ performance across countries that can be influenced by the effort students invest in preparing PISA assessment, a variable “difference in test effort (or relative test effort)” is used. This based on the “Effort Thermometer” that was developed by a group of researchers at the Max–Planck-Institute in Berlin (Kunter et al. 2002). The Effort Thermometer is based on three 10-point scales (For more details, see Butler and Adams 2007) Effort Difference = PISA Effort − School Mark Effort The Effort Difference scores can range from negative nine to positive nine. A negative score on Effort Difference means that students indicate they would try harder on a test that counts than they did on the PISA assessment
Out of school study time	The index was calculated by summing the time spent studying for school subjects from the information that how much time they spent studying outside school (in open-ended format)
Perseverance	Five items measuring perseverance (i.e. a). When confronted with a problem, I give up easily, (b) I put off difficult problems, (c) I remain interested in the tasks that I start, (d) I continue working on tasks until everything is perfect, and (e) When confronted with a problem, I do more than what is expected for me) were included with five response categories, namely 1. Very much like me 2. Mostly like me 3. Somewhat like me 4. Not much like me 5. Not at all like me All three items were reversed
Perceived control	The index of perceived control is constructed using student responses on question “what you think that you can succeed with enough effort (or the course material is too hard to understand with your sole effort)? Students give responses that they strongly agreed, agreed, disagreed, or strongly disagreed
Instrumental motivation for job and career	The index of instrumental motivation for job and career is constructed by asking question that making an effort is worthwhile for me because it will increase chances to get a job and will improve my career with student responses over the extent they strongly agreed, agreed, disagreed, or strongly disagreed
Subjective norms (Mathematics)	The index of subjective norms in mathematics is constructed using student responses over whether, thinking about how people important to them view mathematics, they strongly agreed, agreed, disagreed or strongly disagreed to the following statements: Most of my friends do well in mathematics; most of my friends work hard at mathematics; my friends enjoy taking mathematics tests; my parents believe it’s important for me to study mathematics; my parents believe that mathematics is important for my career; my parents like mathematics

**Table 3:** Structure of the global gender gap index, 2012. Source: The Global Gender Gap Report 2012, World Economic Forum

Subindex	Variable	Standard deviation	Weights
Economic participation and opportunity	Ratio of female to male labour force participation rate Wage equality ratio between women and men for similar work Estimated female to male earned income Female to male value for legislators, senior officials and managers Female to male value for professionals and technical workers	0.160 0.103 0.144 0.214 0.262	0.199 0.310 0.221 0.149 0.121
Educational attainment	Ratio of female to male literacy rate Ratio of female to male net primary level enrolment Ratio of female to male net secondary level enrolment Ratio of female to male net tertiary level enrolment	0.145 0.060 0.120 0.228	0.191 0.459 0.230 0.121
Health and survival	Female to male sex ratio at birth Female to male health life expectancy ratio	0.023 0.010	0.307 0.693
Political empowerment	Female to male seats in parliament ratio Female to male ministerial level ratio value Female to male ratio for number of years of a female head of state or government	0.166 0.208 0.116	0.310 0.247 0.443

The Global Gender Gap Index examines the gap between male and female in four fundamental categories (subindexes). The four subindexes are divided into 14 different variables to compose them. Weights are assigned for each variable according to the rule of same relative impact on the subindex. A variable with a small variability (or standard deviation) gets a larger weight within that subindex. All variables within each sub-index adds to one. GGI, 2012 is the average of the four subindices, ranging from 0 to 1 with a max value of 0.86 for Iceland and minimum value of 0.50 for Yemen

**Table 4:** Descriptive statistics

Variable	Mean	Std. dev	Minimum value	Maximum value	No. of obs.
*Test scores*					
***Math scores***					
*Math scores (girls)*	*469.1*	*102.6*	*19.8*	*912.3*	*242375*
*Math scores (boys)*	*478.8*	*107.5*	*34.7*	*962.2*	*237799*
*Math (OECD)*	*494.05*	*104.7*	*59.67*	*896.8*	*295,416*
Math scores (girls, OECD)	488.2	101.7	98.23	896.8	147,717
Math scores (boys, OECD)	499.8	107.1	59.67	891.3	147,699
*Math (Non-OECD)*	*451.93*	*98.6*	*19.79*	*962.2*	*184,758*
Math scores (Girls, Non-OECD)	448.6	96.8	19.79	912.3	94,658
Math scores (Boys, Non-OECD)	455.3	100.3	34.75	962.2	90,100
***Reading scores***					
*Reading scores (Girls)*	*493.08*	*97.93*	*0.0834*	*904.8*	*242,375*
*Reading scores (Boys)*	*455.85*	*105.7*	*0.0834*	*889.3*	*237,799*
*Reading (OECD)*	*496.4*	*105.9*	*2.546*	*904.8*	*295,416*
Reading (Girls, OECD)	515.2	99.2	2.546	904.8	147,717
Reading (Boys, OECD)	478.1	108.2	3.911	889.3	147,699
*Reading (Non-OECD)*	*450.3*	*94*	*0.083*	*838.7*	*184,758*
Reading (Girls, Non-OECD)	469.3	89.1	0.083	836.8	94,658
Reading (Boys, Non-OECD)	431	95.6	0.083	838.7	90,100
*Math sub-scale (content) scores*					
Change and relationship (OECD)	*492.6*	*115.7*	*10.68*	*941.9*	*295,416*
Change and relationship (Girls, OECD)	487.0	111.9	10.68	873.9	147,717
Change and relationship (Boys, OECD)	498.2	118.9	17.46	941.9	147,699
*Change and relationship (Non-OECD)*	*448.4*	*110*	*6.551*	*980.8*	*184,758*
Change and relationship (Girls, Non-OECD)	446.1	107.7	6.551	968.7	94,658
Change and relationship (Boys, Non-OECD)	450.9	112.1	6.551	980.8	90,100
***Quantity (OECD)***	*495.2*	*110*	*20.42*	*885.5*	*295,416*
Quantity (Girls, OECD)	489.6	107.2	20.42	855.5	147,717
Quantity (Boys, OECD)	500.8	112.3	26.88	885.5	147,699
***Quantity (Non-OECD)***	*448.2*	*103.2*	*3.357*	*934.9*	*184,758*
Quantity (Girls, Non-OECD)	444.3	101.3	7.875	907.8	94,658
Quantity (Boys, Non-OECD)	452.2	104.9	3.357	934.9	90,100
***Space*** *** and*** ***shape (OECD*** **)**	*489.7*	*112.6*	*1.254*	*963.2*	*295,416*
Space and shape (Girls, OECD)	481.6	110	13.33	917.8	147,717
Space and shape (Boys, OECD)	497.6	114.3	1.254	963.2	147,699
Space and shape (Non-OECD)	*456.6*	*108.1*	*3.980*	*1082.1*	*184,758*
Space and shape (Girls, Non-OECD)	450.9	107.2	3.980	1022.1	94,658
Space and shape (Boys, Non-OECD)	462.4	108.8	24.47	1082.1	90,100
Uncertainty and data (OECD)	*493.2*	*105.6*	*7.953*	*892.2*	*295,416*
Uncertainty and data (Girls, OECD)	488.6	102.1	7.953	849.7	147,717
Uncertainty and data (Boys, OECD)	497.8	108.7	7.953	892.2	147,699
Uncertainty and data (Non-OECD)	*449.1*	*93.11*	*9.044*	*941.1*	*184,758*
Uncertainty and data (Girls, Non-OECD)	447.8	90.64	9.044	905.3	94,658
Uncertainty and data (Boys, Non-OECD)	450.5	95.46	24.39	941.1	90,100
*Students’ own characteristics*					
***Gender of a student (Reference category is “Male”)***					
Female	0.4998	500	0	1	242,375
Male	0.5	500	0	1	237,799
***Age of the student***					
Age	15.78	0.291	15.17	16.33	480,058
Age (Missing)	0.0001	0.012	0	1	116
Age (After imputation)	15.78	0.291	15.17	16.33	480,174
***Grade in which students are enrolled (reference category: Grade 7 + 8 + 9)***					
Grade 7 + 8 + 9	0.452	0.498	0	1	179,603
Grade 10 + 11 + 12 + 13	0.546	0.498	0	1	299,698
Ungraded	0.0025	0.0504	0	1	873
***Country of birth (Reference category is “Other country”)***					
Country of test	0.915	0.2783	0	1	437,025
Other country	0.066	0.248	0	1	34,126
N/A + Invalid + Missing	0.019	0.135	0	1	9023
*Family characteristics*					
***Mothers’ Education (Reference category is “None*** + ***Primary*** + ***Lower secondary*** + ***Upper secondary “)***					
None + Primary + Lower secondary + Upper secondary	0.32	0.466	0	1	157,228
Post secondary + Tertiary (Bachelor) + Tertiary (Master, Doctoral)	0.641	0.48	0	1	304,692
Missing	0.04	0.195	0	1	18,254
***Fathers’ Education (Reference category is “None*** + ***Primary*** + ***Lower secondary*** + ***Upper secondary “)***					
None + Primary + Lower secondary + Upper secondary	0.32	0.467	0	1	158,477
Post secondary + Tertiary (Bachelor) + Tertiary (Master, Doctoral)	0.611	0.488	0	1	289,539
Missing	0.069	0.253	0	1	32,158
***Mothers’ work status (Reference category is “Part-time*** + ***Looking for a job*** + ***other”)***					
Mothers working full time	0.4701	0.499	0	1	215,933
Part-time + Looking for a job + Other	0.492	0.5	0	1	246,620
N/A + Invalid + Missing	0.038	0.191	0	1	17,621
***Fathers’ work status (Reference category is “Part-time*** + ***Looking for a job*** + ***other”)***					
Fathers working full time	0.6995	0.458	0	1	335,802
Part-time + Looking for a job + Other	0.229	0.42	0	1	110,709
N/A + Invalid + Missing	0.072	0.258	0	1	33,663
***Family structure (Reference category is “Single parent”)***					
Single parent	0.121	0.326	0	1	57,304
Two parents	0.74	0.439	0	1	356,286
Other + Missing	0.139	0.346	0	1	66,584
***International language at home (Reference category is “Other language”)***					
Language of the test	0.852	0.355	0	1	403,608
Other language	0.108	0.311	0	1	56,687
N/A + Invalid + Missing	0.039	0.194	0	1	19,879
School/class characteristics					
***School category (Reference category is “Private school”)***					
Public school	0.797	0.403	0	1	378,889
Private school	0.184	0.388	0	1	93,041
N/A + Invalid + Missing	0.019	0.137	0	1	8244
***School autonomy***					
School autonomy	− 0.072	1.057	− 2.872	1.604	470,968
Missing	0.021	0.142	0	1	9206
School autonomy (Imputed)	− 0.072	1.046	− 2.872	1.604	480,174
***Class size***					
Class size	29.06	9.42	13	53	462,865
Missing	0.03	0.17	0	1	17,309
Class size (Imputed)	29.06	9.28	13	53	480,174
***Quality of physical infrastructure***					
Quality of physical infrastructure	− 0.118	1.048	− 2.76	1.305	461,829
Missing	0.042	0.202	0	1	18,345
Quality of physical infrastructure(Imputed)	− 0.118	1.026	− 2.76	1.305	480,174
***Proportion of girls at school***					
Proportion of girls at school	0.488	0.196	0	1	465,353
N/A + Invalid + Missing	0.037	0.189	0	1	14,821
Proportion of girls at school(Imputed)	0.488	0.193	0	1	480,174
***Proportion of certified teachers at school***					
Proportion of certified teachers at school	0.865	0.275	0	1	376,980
N/A + Invalid + Missing	0.183	0.387	0	1	103,194
Proportion of certified teachers at school (Imputed)	0.865	0.249	0	1	480,174
*Students’ perceptions or beliefs about learning*					
***Difference in test effort = (PISA Effort – School Mark Effort) (Reference category is “Positive test effort”)***					
Difference in test effort excluding N/A, Invalid and Missing	0.873	0.333	0	1	418,698
N/A + Invalid + Missing	0.127	0.333	0	1	61,476
Difference in test effort (Negative or zero)	0.276	0.447	0	1	127,858
Difference in test effort (Positive)	0.597	0.490	0	1	290,840
***Out of school study time***					
Out of school study time (Excluding missing)	11.11	10.58	0	180	305,317
Missing	0.365	0.481	0	1	174,857
Out of school study time (Imputed)	11.11	8.431	0	180	480,174
***Perseverance(willingness to work on problems that are difficult)***					
Perseverance (Excluding missing)	0.124	0.995	− 4.053	3.529	309,742
Missing	0.356	0.479	0	1	170,432
Perseverance (Imputed)	0.124	0.797	− 4.053	3.529	480,174
**Perceived control (can succeed with enough effort) (Reference category is “Disagree, Strongly disagree”)**					
Strongly agree	0.308	0.462	0	1	153,582
Agree	0.291	0.454	0	1	136,272
Disagree, Strongly disagree	0.048	0.214	0	1	21,667
N/A + Invalid + Missing	0.353	0.478	0	1	168,653
***Instrumental motivation (good for career chances) (Reference category is “Disagree, Strongly disagree”)***					
Strongly agree	0.195	0.396	0	1	98,403
Agree	0.315	0.465	0	1	151,583
Disagree, Strongly disagree	0.137	0.344	0	1	61,790
N/A + Invalid + Missing	0.353	0.478	0	1	168,398
***Instrumental motivation (good to get a job) (Reference category is “Disagree, Strongly disagree”)***					
Strongly agree	0.167	0.373	0	1	84,270
Agree	0.306	0.461	0	1	148,611
Disagree, Strongly disagree	0.174	0.379	0	1	78,895
N/A + Invalid + Missing	0.352	0.478	0	1	168,398
***Country characteristics***					
Non-OECD	0.4770	0.499	0	1	184,758
OECD	0.5230	0.499	0	1	295,416
GDP per capita (PPP)	33,937.2	24,246.2	5000.8	135,421.7	479,881
GGI	0.7108	0.0544	0.6015	0.864	452,918
Gender ratio at PISA test	1.019	0.0854	0.84	1.3	478,413
Expenditures on education (%)	5.0116	1.372	2.1	8.7	450,865

**Table 5:** International gender gap in math and reading test scores at various percentiles

	1st	5th	10th	25th	50th = median	Std. dev.	75th	90th	95th	99th
*Test score performance in mathematics*										
Girls’ scores	248.8	305.51	336.66	391.97	460.59	100.17	532.96	596.60	632.74	698.17
Boys’scores	246.31	307.14	339.78	398.75	472.12	106.16	549.39	616.30	652.99	717.64
Gender gap = (Girls–Boys)	2.49	− 1.63	− 3.12	− 6.78	− 11.53		− 16.43	− 19.7	− 20.25	− 19.47
*Test score performance in reading*										
Girls’ scores	257.36	327.42	363.88	424.48	491.84	96.49	556.97	612.02	643.32	698.28
Boys’scores	201.99	276.09	315.95	381.95	456.29	105.22	528.46	587.81	620.13	678.35
Gender gap = (Girls–Boys)	55.37	51.33	47.93	42.53	35.55		28.51	24.21	23.19	19.93

Firstly, we calculated the performance percentiles for girls and boys separately for each assessment and then for each assessment, we calculated the gender differences in performance distribution by subtracting the boys’ scores from girls’ scores

**Table 6:** Juhn–Murphy–Pierce decomposition of mathematics test scores by gender

Percentiles	T	Q	P	U
*Individual characteristics*				
p5	0.6232	− 1.6601	10.627	− 8.3435
p10	1.7136	− 2.6240	11.1438	− 6.8062
p25	4.7516	− 3.0524	11.534	− 3.7298
p50	9.2694	− 2.7514	11.829	0.1914
p75	14.644	− 1.8629	12.234	4.2733
p90	17.916	− 1.4133	12.525	6.8035
p95	18.928	− 1.3949	13.001	7.3219
*Family characteristics*				
p5	0.6232	0.3782	9.3689	− 9.1239
p10	1.7136	− 0.8306	9.6578	− 7.1136
p25	4.7516	− 1.2217	9.9602	− 3.9869
p50	9.2694	− 0.3162	9.6565	− 0.0709
p75	14.644	0.9631	9.5052	4.1756
p90	17.916	0.4696	9.8460	7.6000
p95	18.928	0.0490	9.7870	9.0921
*Schooling characteristics*				
p5	0.6232	− 5.9810	15.933	− 9.3289
p10	1.7136	− 7.2584	16.637	− 7.6655
p25	4.7516	− 7.8643	17.044	− 4.4281
p50	9.2694	− 7.6651	16.846	0. .0887
p75	14.644	− 7.4658	17.388	4.7218
p90	17.916	− 7.7754	17.839	7.8517
p95	18.928	− 8.8790	19.016	8.7914
*Belief characteristics*				
p5	0.6232	− 3.4441	8.5159	− 4.4486
p10	1.7136	− 4.1805	10.000	− 4.1060
p25	4.7516	− 4.1604	11.559	− 2.6474
p50	9.2694	− 2.4598	12.256	− 0.5264
p75	14.644	− 1.5339	13.877	2.3006
p90	17.916	− 1.3770	14.394	4.8983
p95	18.928	− 2.1631	15.337	5.7538
*Country characteristics*				
p5	0.0779	2.2793	6.9492	− 9.1506
p10	1.1684	1.2424	7.5482	− 7.6222
p25	4.3621	1.1179	7.8306	− 4.5864
p50	8.2568	0.8006	7.8884	− 0.4322
p75	14.021	1.5481	7.8875	4.5853
p90	17.215	0.95,581	7.8215	8.4372
p95	18.227	0.6335	7.6553	9.9383
*All characteristics*				
p5	0.0779	− 10.119	14.258	− 4.0610
p10	1.1684	− 11.203	15.762	− 3.3903
p25	4.3621	− 10.737	17.339	− 2.2400
p50	8.2568	− 8.1646	16.836	− 0.4148
p75	14.021	− 6.0930	18.046	2.0675
p90	17.215	− 5.4332	18.752	3.8954
p95	18.227	− 6.4822	19.588	5.1214

**Table 7:** Juhn–Murphy–Pierce decomposition of reading test scores by gender

Percentiles	T	Q	P	U
*Individual characteristics*				
p5	− 51.798	− 1.9803	− 36.400	− 13.419
p10	− 49.510	− 2.8214	− 35.864	− 10.825
p25	− 44.506	− 3.4393	− 35.142	− 5.9239
p50	− 37.592	− 3.0823	− 34.603	0.0927
p75	− 30.392	− 2.1495	− 34.424	6.1816
p90	− 25.133	− 1.2906	− 33.860	10.018
p95	− 23.075	− 1.4792	− 33.746	12.150
*Family characteristics*				
p5	− 51.798	0..6290	− 37.766	− 14.662
p10	− 49.510	− 0.8843	− 36.781	− 11.845
p25	− 44.506	− 1.9307	− 36.540	− 6.0346
p50	− 37.592	− 0.8760	− 36.929	0.2126
p75	− 30.392	0.5776	− 37.222	6.2526
p90	− 25.133	1.4306	− 37.325	10.762
p95	− 23.075	0.9785	− 37.339	13.286
*Schooling characteristics*				
p5	− 51.798	− 6.9977	− 31.159	− 13.642
p10	− 49.510	− 7.9886	− 29.939	− 11.583
p25	− 44.506	− 9.2939	− 28.970	− 6.2421
p50	− 37.592	− 9.0559	− 28.759	0..2229
p75	− 30.392	− 8.3132	− 28.208	6.1292
p90	− 25.133	− 8.4905	− 27.146	10.503
p95	− 23.075	− 10.373	− 25.693	12.992
*Belief characteristics*				
p5	− 51.798	− 4.7127	− 37.495	− 9.5907
p10	− 49.510	− 4.2156	− 36.305	− 8.9900
p25	− 44.506	− 3.5509	− 34.893	− 6.0617
p50	− 37.592	− 2.1595	− 34.672	− 0.7610
p75	− 30.392	− 1.6176	− 33.969	5.1950
p90	− 25.133	− 1.7336	− 33.013	9.6143
p95	− 23.075	− 2.4504	− 32.282	11.657
*Country characteristics*				
p5	− 51.648	1.8113	− 38.138	− 15.321
p10	− 48.883	1.0150	− 37.919	− 11.979
p25	− 44.276	0.6254	− 38.238	− 6.6628
p50	− 38.243	0.4795	− 38.578	− 0.1441
p75	− 30.563	1.3517	− 39.038	7.1231
p90	− 25.540	1.5354	− 39.435	12.360
p95	− 23.638	0.6097	− 39.488	15.240
*All characteristics*				
p5	− 51.648	− 11.752	− 31.189	− 8.7071
p10	− 48.883	− 12.374	− 29.587	− 6.9220
p25	− 44.276	− 12.192	− 28.067	− 4.0162
p50	− 38.243	− 9.9101	− 27.790	− 0.54,317
p75	− 30.563	− 7.1428	− 27.426	4.0055
p90	− 25.540	− 6.4802	− 26.577	7.5171
p95	− 23.638	− 7.6703	− 25.447	9.4795

## Footnotes

1 The first PISA exam in 2000 focused on reading literacy, while the second focused on mathematics specialization. PISA 2012 again focused on mathematics literacy.

2 The PISA consortium decides which school will participate, and then the school provides a list of eligible students. Students are selected by national project managers according to standardized procedures (OECD 2012).

3 These plausible values are calculated by the complex item-response theory (IRT) model (see Baker 2001; Von Davier and Sinharay 2013) based on the assumption that each student only answers a random subset of questions and their true ability cannot be directly judged but only estimated from their answers to the test. This is a statistical concept, and instead of obtaining a point estimate [like a Weighted Likelihood Estimator (WLE)], a range of possible values of students’ ability with an associated probability for each of these values is estimated (OECD 2009).

4 “Working with one plausible value instead of five provides unbiased estimates of population parameters but will not estimate the imputation error that reflects the influence of test unreliability for the parameter estimation” (OECD 2009). As this imputation error decreases with a large sample size, so the use of one plausible value with a sample size of 480,174 students will not make any substantial difference in the mean estimates and standard errors of the estimates. For details, see p 43: https://www.oecd-ilibrary.org/docserver/9789264056275-en.pdf?expires=1537249103&id=id&accname=guest&checksum=FCF6D3D8A03AB42A0FEC82FE7E2ADF47.

5 The detailed structure of GGI is provided in Table 2 in Appendix.

6 GGI data for Liechtenstein, Montenegro, and Tunisia is unavailable.

7 See Munir (2017) for details.

8 Other methods, like Machado and Mata (2005) provide a similar decomposition, extending the Blinder-Oaxaca framework along quantiles, Juhn et al. (1993) have the advantage that they also provide for a distribution of residuals.

9 These outcomes are actually equal to the originally observed values, i.e., ${\text{y}}_{\text{ij}}^{( 3)} = {\text{ y}}_{\text{ij}} = {\text{ x}}_{\text{ij}} \beta_{\text{j}} + \, \varepsilon_{\text{ij}}$.

10 These are school autonomy, class size, quality of physical infrastructure, proportion of girls at school, out of school study time and perseverance.

11 These results are not presented here because of space limitations but are available upon request.

12 See also Stoet and Geary (2013) for the inverse relationship between mathematics and reading assessments.
